# Prevalence, correlates and solutions to people with HIV in China being refused treatment for diseases not related to HIV: a mixed‐methods study

**DOI:** 10.1002/jia2.26443

**Published:** 2025-04-11

**Authors:** Yuqing Hu, Xinyi Zhou, Xinxue Fan, Rongjun Bi, Yingqi Deng, Hui Li, Xin Peng, Dan Luo, Heping Zhao, Zhihui Guo, Longtao He, Huachun Zou

**Affiliations:** ^1^ School of Public Health (Shenzhen) Sun Yat‐sen University Shenzhen China; ^2^ Shizhong District Center for Disease Control and Prevention Jinan China; ^3^ School of Public Health, Fudan University Shanghai China; ^4^ Research Institute of Social Development, Southwestern University of Finance and Economics Chengdu China; ^5^ School of Public Health, Southwest Medical University Luzhou China; ^6^ Kirby Institute, University of New South Wales Sydney New South Wales Australia

**Keywords:** China, epidemiology, health policy, people with HIV, public health, treatment refusal

## Abstract

**Introduction:**

Existing studies on treatment refusal towards people with HIV (PWH) lack focus on the Chinese context and key factors. We aimed to elucidate the prevalence, correlates and solutions to PWH being refused treatment for diseases not related to HIV (DNRH) in China.

**Methods:**

We conducted a mixed‐methods study of PWH and healthcare providers (HCPs) between April 2021 and June 2022. An online survey of PWH assessed the prevalence and correlates of treatment refusal for DNRH during their most recent outpatient or inpatient visit. Semi‐structured telephone interviews were conducted with PWH and HCPs to understand their experiences of treatment refusal and to generate potential solutions.

**Results:**

We included 35 PWH and 30 HCPs in the interviews, and 902 PWH in the survey. In the survey, 42.2% and 63.0% of PWH reported treatment refusal for DNHR during their most recent outpatient and inpatient visit, respectively. Among outpatients, PWH who were <30 years old (AOR: 0.43, 95% CI: 0.25−0.73), acquired HIV through male‐male sex (0.56, 0.35−0.90) and did not disclose their HIV status to HCPs (0.64, 0.42−0.96) were less likely to report treatment refusal. PWH who were not adherent to antiretroviral therapy (10.66, 1.16−98.20), had their outpatient visit before the COVID‐19 pandemic (1.74, 1.00−3.00) and received care at a surgical department (2.10, 1.23−3.60) were more likely to report treatment refusal. Among inpatients, PWH who received care from a male HCP (2.31, 1.27−4.22) and were hospitalized in central provinces of China (2.60, 1.07−6.31) had higher odds of treatment refusal. In semi‐structured interviews, we found HCP refusal to treat PWH for DNRH could be influenced by stigma against HIV, concerns about HIV acquisition, limited knowledge of HIV post‐exposure prophylaxis and insufficient protection from health authorities against discrimination by HIV status. Participants identified several solutions that may help mitigate treatment refusal, including supporting PWH to achieve virologic suppression, HIV education for HCPs, employment protections and compensation for HCPs who acquire HIV in the workplace, and the establishment of dedicated government offices and laws to address treatment refusal.

**Conclusions:**

Treatment refusal for DNRH was common among PWH in China. Factors contributing to treatment refusal involve PWH, HCPs and health authorities. Systematic interventions involving all stakeholders, particularly legal protections against discrimination by HIV status, should be implemented to reduce treatment refusal.

## 1 INTRODUCTION

Antiretroviral therapy (ART) dramatically improved the prognosis and life expectancy of people with HIV (PWH) [1, 2]. As life expectancy has improved, a growing proportion of PWH are experiencing diseases not related to HIV (DNRH), including non‐AIDs‐defining cancers and cardiovascular diseases [3, 4, 5]. These trends pose a substantial challenge to healthcare globally, with the need to optimize resources, treat patients and improve overall healthcare among PWH [1, 2]. Numerous studies have reported that PWH frequently experience treatment refusal in low‐ and middle‐income countries, including India [6], China [7], Iran [8] and Ethiopia [9]. Healthcare providers (HCPs) refusing to treat PWH not only has a detrimental effect on physical health, but also negatively affects the happiness, self‐esteem and long‐term mental health of PWH [10].

In China, treatment refusal towards PWH presents a complex challenge. In 2004, the Chinese government designated specific hospitals as specialized sites for the provision of care to PWH. These facilities have more knowledge and experience in providing HIV care and are less likely to refuse treatment for PWH [7]. However, their capability to treat DNRH is limited, driving PWH to seek medical care for conditions besides HIV at other facilities [7]. Regrettably, in clinics or hospitals that do not specialize in HIV care, PWH in China may face treatment refusal due to their HIV status [11]. This highlights the critical need to raise awareness about treatment refusal to guarantee that all PWH receive necessary medical care irrespective of the hospital or clinic to which they present [12].

While previous studies have investigated HCPs refusal to provide treatment to PWH [6, 8, 9, 10, 13], these studies were conducted in countries with different healthcare systems than China. Therefore, findings from previous research may not be directly applicable to the Chinese context. Moreover, previous studies have predominately focused on the perspectives of HCPs [7, 11], neglecting other factors (e.g. socio‐demographic characteristics of PWH, different departments within hospitals), which might also correlate with treatment refusal. The use of a single method (i.e. quantitative or qualitative) in these studies has also limited previous investigations of treatment refusal. To address these gaps, we conducted a nationwide mixed‐methods study involving both PWH and HCPs to better understand the prevalence, correlates and solutions to PWH in China being refused treatment for DNRH.

## 2 METHODS

### 2.1 Study design and data collection

**Figure 1:**
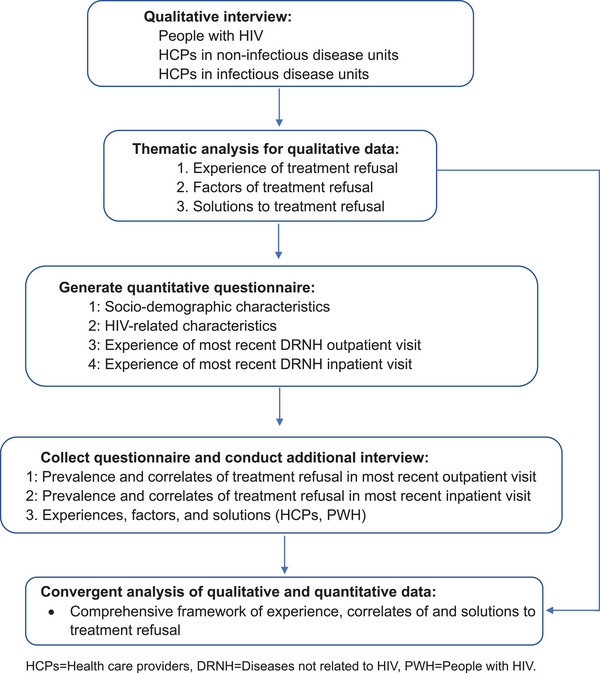
Flowchart of the mixed‐methods study. DRNH, diseases not related to HIV; HCPs, healthcare providers; PWH, people with HIV.

This mixed‐methods study was conducted between April 2021 and June 2022. First, we conducted semi‐structured one‐on‐one online interviews with PWH as well as HCPs from infectious and non‐infectious disease departments to explore experiences with treatment refusal, potential factors contributing to treatment refusal (e.g. the medical department involved, HCPs’ gender, geographic region, etc.) and possible solutions. Then, an online survey was generated and used to collect quantitative data on socio‐demographic characteristics and treatment refusal among PWH. We performed additional interviews to identify the comprehensive framework of experiences, correlates of and solutions to treatment refusal for DNRH. The study design is summarized in Figure 1.

Qualitative online interviews were conducted with PWH and HCPs employed at hospitals. A purposive sampling strategy was employed to recruit eligible participants, with the objective of achieving diverse representation across a range of demographic characteristics, HIV‐related attributes and medical experiences with DNRH following HIV acquisition. We recruited PWH across a range of socio‐demographic categories, including age, gender, educational attainment, income level, transmission route, time since HIV acquisition and current HIV viral load. Participants were also purposely selected to encompass a range of DNRH medical experiences, including outpatient visits, inpatient admissions, surgical procedures and internal medicine consultations. Following the conclusion of the purposive sampling strategy, Dr Li Hui directed potential eligible PWH to our research team, all of whom are her patients. With nearly four decades of experience in the HIV field, Dr Li Hui has provided assistance to a considerable number of PWH and is the founder of “Li Hui Shi Kong,” a widely followed HIV‐focused WeChat account in China [14, 15], with over 100,000 PWH followers. Following the referral from Dr Li Hui, we proceeded to contact the potential participants directly via WeChat. This initial contact was used to ascertain their eligibility, informed consent and then schedule interviews.

We purposively invited HCPs from diverse backgrounds, including age, gender, educational background, professional designation, departmental affiliation and experience with HIV care to participate in our interviews. HCPs were recruited through our professional networks. The corresponding author (HZ) has been conducting research in the HIV field since 2014, and the first author (YH) has completed a year and a half of clinical training, allowing our team to be familiar with a wide network of HCPs in China. Informed consent was obtained via WeChat, and semi‐structured one‐to‐one online interviews were conducted via WeChat voice calls.

The Interpretive Phenomenological Analysis approach was selected as the methodological framework for conducting interviews and analysing transcripts [16]. Interviews examined the experiences regarding treatment refusal for DNRH, including contributing factors and participant suggestions for strategies to mitigate treatment refusal. Interview guides are provided in Tables S1 and S2. Interviews were conducted by trained researchers, lasted approximately 30 minutes and were audio‐recorded. We used an iterative approach where data were analysed as they were collected. Two researchers independently analysed interview responses, and then compared findings with the other researcher to reach a consensus. This collaborative approach was designed to reduce the influence of individual bias and facilitate a more comprehensive identification of themes. Analytic memos were produced by each analyst, which documented the evolution of themes over time. This approach enabled the rapid identification of emerging themes and critical reflections, which in turn informed the subsequent data collection process, allowing for the refinement of underexplored concepts. Recruitment and interviews continued until information saturation was reached, where no new themes, concepts or categories emerged despite continued data collection and analysis [17]. A remuneration of CNY 50 (approximately USD 7.0) was provided to each participant.

PWH were eligible to complete the online quantitative survey irrespective of their DNRH medical history. A publicly available link to the survey was disseminated on the “Li Hui Shi Kong” WeChat account. Informed consent was obtained prior to the commencement of the survey. The survey collected information on socio‐demographic characteristics (e.g. age, gender, highest level of education, monthly income, marital status, occupation), HIV‐related characteristics (e.g. time since HIV acquisition, transmission route, use of ART and HIV viral load), and most recent DNRH medical experiences following HIV acquisition in outpatient and inpatient settings. Questions about DNRH medical experiences collected information on treatment refusal, time, location, and department of outpatient and inpatient visits, as well as the gender of the HCP and the type of hospital. Surveys are available in the Supplementary Material. To be valid, survey respondents needed to provide correct responses to validation questions, such as correctly identifying the capital city of China, and completing the whole questionnaire in more than 120 seconds.

We aimed to complete a total of at least 451 survey responses. This sample size was calculated using the cross‐sectional sample size calculation equation [18], with a precision of 0.05 and two‐sided α = 0.05. We assumed the proportion of respondents who had experienced treatment refusal would be approximately 47% based on previous research in Dalian, China [19]. To account for potential missing or incomplete data, we increased our recruitment goal to 500 eligible respondents.

In the initial round of qualitative interview recruitment, there was some uncertainty as to whether the interviewees had also completed the quantitative survey. However, it was probable that they had, given that all interviewees were recruited solely through Dr. Li Hui's direct referral. To ensure that the qualitative data captured dynamic, expanded explanations or even divergent perspectives from the quantitative data, we conducted additional qualitative interviews, specifically recruiting from quantitative survey participants. Additional interviews were conducted with HCPs who met the same inclusion criteria as those in the initial round of qualitative interviews. These steps facilitated the comprehensive integration of both qualitative and quantitative insights within a mixed‐methods approach.

### 2.2 Data analysis

For qualitative interviews, audio recordings were transcribed verbatim and analysed using NVivo 11.0 (QSR International, Melbourne, Australia). Thematic analysis was performed to identify potential themes related to treatment refusal among PWH. We followed a six‐step analytical process recommended by a previous study [20]: (1) developing familiarity with data, (2) generating initial codes, (3) searching for themes, (4) reviewing themes, (5) defining and naming themes, and (6) producing the report. We generated a list of themes through a comprehensive review of previous relevant publications. Two coders (YH and XZ) independently reviewed and delineated the themes that emerged from our interviews. We calculated the Cohen's Kappa coefficient to be 0.77, indicating good intercoder agreement. Any discrepancies were cross‐referenced with the original theme list and discussed between the two coders. In instances of persistent discordance between the two coders, we sought the input of a qualitative specialist (LH) to facilitate a final resolution. All investigators agreed on the key overarching themes that emerged from the interviews.

Quantitative data was summarized using descriptive statistics. For continuous variables, cutoff points were determined in consultation with clinical experts, taking into account clinical observations, the data distribution of the data and findings from previous literature [15]. Our approach involved three key steps: First, we consulted clinical experts, as they have the most direct experience with this population and could provide cutoff values that are practically meaningful. Second, we reviewed the existing literature to ensure that the suggested cutoff values were scientifically supported. Lastly, we assessed the distribution of the categorised variables by examining the sample sizes within each group, ensuring that no group was excessively small, which could otherwise compromise the stability of our models. Based on these cutoff values, we categorized the variables to provide a clearer representation of their distribution within the sample. We then calculated and reported the numbers and percentages for each category in tabular form. Chi‐square tests were employed to compare socio‐demographic characteristics, and HIV‐related factors, between initial interview participants and survey respondents. Univariable and multivariable logistic regression models were employed to ascertain the correlates of treatment refusal, with variables deemed statistically significant at *p* < 0.05. All data were analysed using the R software (version 4.0.1; R Core Team, Vienna, Austria).

## 3 RESULTS

In total, 30 PWH and 27 HCPs participated in the initial semi‐structured interviews, and 902 PWH completed the online survey (Table 1). The median age of PWH in the initial interviews was 34.0 years old (IQR 27.0−45.5), while that of survey participants was 31.7 years old (IQR 26.7−38.4). The participants represented a diverse range of socio‐economic backgrounds, with most PWH having attained a college degree. Participants displayed a range of HIV‐related characteristics and diverse DNRH medical experiences in both the qualitative and quantitative portions of the study. Most participants were men, comprising 83% of those interviewed and 90.6% of survey respondents. The majority of interview and survey participants reported acquiring HIV through male‐male sex (67% and 81%, respectively) and had undetectable HIV viral loads (93% and 79%, respectively). In the survey, 41% (372/902) of PWH reported having DNRH. No statistically significant differences were observed between the interview and survey participants in terms of socio‐demographic characteristics and HIV‐related factors, with the exception of gender. Specifically, 7.3% of survey respondents identified as transgender, and no transgender individuals were interviewed (*p* < 0.001).

**Table 1:** Characteristics of study participants

People with HIV				
	Initial interview participants (*n* = 30)	Survey participants (*n* = 902)	*p* value	Second set interview participants (*n* = 5)
**Socio‐demographic characteristics**				
Age (years)			*0.38*	
<30	11 (36.7%)	360 (39.9%)		2 (40%)
30–39	10 (33.3%)	205 (22.7%)		2 (40%)
≥40	9 (30%)	337 (37.4%)		1 (20%)
Gender			***<0.001***	
Men	25 (83.3%)	817 (90.6%)		4 (80%)
Women	5 (16.7%)	19 (2.1%)		1 (20%)
Transgender	0	66 (7.3%)		0
Education			*1*	
College degree or higher	23 (76.7%)	695 (77.1%)		4 (80%)
Below college degree	7 (23.3%)	207 (22.9%)		1 (20%)
Monthly income (CNY)			*0.31*	
<5000	9 (30.0%)	390 (43.2%)		2 (40%)
5000–10,000	15 (50.0%)	340 (37.7%)		2 (40%)
>10,000	6 (20.0%)	172 (19.1%)		1 (20%)
Comorbidities			−	
CVD	−	61 (6.8%)		−
Cancer	−	18 (2.0%)		−
Chronic respiratory diseases	−	92 (10.4%)		−
Other chronic diseases	−	18 (2.0%)		−
Other IDDs	−	92 (10.2%)		−
Other STDs	−	101 (11.2%)		−
None of these diseases	−	530 (58.8%)		−
**HIV‐related characteristics**				
Transmission route			*0.10*	
Male‐male sex	20 (66.7%)	726 (80.5%)		4 (80%)
Male‐female sex or other	10 (33.3%)	176 (19.5%)		1 (20%)
Years since HIV acquisition			*0.34*	
<3	3 (10%)	96 (10.6%)		1 (20%)
≥3, <6	10 (33.3%)	414 (45.9%)		2 (40%)
≥6	17 (56.7%)	392 (43.5%)		2 (40%)
HIV viral load			*0.10*	
Undetectable	28 (93.3%)	716 (79.4%)		4 (80%)
Detectable or unknown	2 (6.7%)	186 (20.6%)		1 (20%)
**DNRH medical experiences after HIV acquisition**				
Most recent outpatient visit			−	
Experienced refusal	10 (33.3%)	212 (23.5%)		2 (40%)
Did not experience refusal	14 (46.7%)	290 (32.2%)		3 (60%)
Not applicable^a^	6 (20.0%)	400 (44.3%)		0 (0%)
Most recent inpatient visit			−	
Experienced refusal	13 (43.3%)	165 (18.3%)		1 (20%)
Did not experience refusal	3 (10.0%)	97 (10.8%)		2 (40%)
Not applicable^a^	14 46.7%)	640 (71.0%)		2 (40%)

Healthcare providers				
	Initial interview		Second set interview	
	Infectious disease units (*n* = 10)	Non‐infectious units (*n* = 17)	Infectious disease units (*n* = 1)	Non‐infectious units (*n* = 2)
Age, years old				
<30	1 (10%)	5 (29.41%)	0 (0%)	1 (50%)
30–39	5 (50%)	7 (41.18%)	0	0
≥40	4 (40%)	5 (29.41%)	1 (100%)	1 (50%)
Gender				
Men	3 (30.0%)	7 (41.2%)	0 (0%)	1 (50%)
Women	7 (70.0%)	10 (58.8%)	1 (100%)	1 (50%)
Transgender	0	0	0	0
Education				
Bachelor's degree or lower	5 (50.0%)	12 (70.6%)	0 (0%)	0 (0%)
Master's degree or higher	5 (50.0%)	5 (29.4%)	1 (100%)	2 (100%)
Title of healthcare providers				
Resident	1 (10.0%)	7 (41.2%)	0 (0%)	1 (50%)
Attending physician	5 (50.0%)	7 (41.2%)	0 (0%)	0 (0%)
Chief physician	4 (40.0%)	3 (17.6%)	1 (100%)	1 (50%)

*Note*: The *p* values were calculated by comparing “Initial interview participants (*n* = 30)” and “Questionnaire participants (*n* = 902).”

Italic values represent *p*‐values obtained from statistical tests. Bold values indicate statistically significant results (*p* < 0.05).

Abbreviations: CVD, cardiovascular disease; DNRH, diseases not related to HIV; IDDs, immunodeficiency diseases; IQR, interquartile range; STDs, sexually transmitted diseases; 1 CNY ≈ 0.14 USD, 22nd October 2024.

^a^ Participants have not sought medical assistance since HIV acquisition.

In the initial interviews (Table 1), 10 (37%) and 17 (63%) HCPs were employed in infectious disease departments and non‐infectious disease departments, respectively. Over half of HCPs were women and held positions at or above the level of attending physician.

To develop a comprehensive framework of experiences, correlates and solutions to treatment refusal for DNRH, an additional set of semi‐structured interviews was conducted with five PWH and three HCPs (Table 1). The characteristics of these individuals are presented in Table 1.

In the initial interviews, 24 (80%) PWH reported having sought medical care for DNRH and 10 (42%) had experienced treatment refusal by HCPs. Additionally, HCPs in both infectious and non‐infectious disease departments reported observing instances of treatment being refused to PWH in their practice settings (Table S3).

### 3.1 Prevalence of treatment refusal and its quantitative correlates

**Figure 2:**
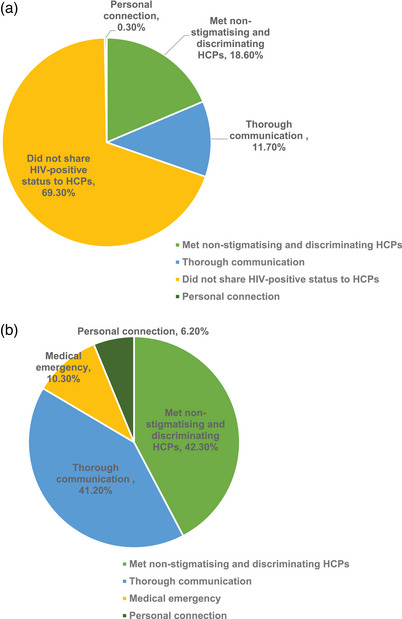
Reasons for PWH's successful outpatient and inpatient visit (a: outpatient visit; b: inpatient visit). HCPs, healthcare providers; PWH, people with HIV.

As illustrated in Table 2, the quantitative survey found that 42% (247/502) of respondents had experienced treatment refusal during their most recent outpatient visit (Table 2). Among those who did not experience treatment refusal, over half (67%, 159/239) identified not sharing their HIV‐positive status with HCPs as the most important reason for not being refused treatment by HCPs (Figure 2a). Table 2 presents the results of the multivariable logistic regression model. PWH who were less than 30 years old (AOR 0.43, 95% CI: 0.25−0.73), acquired HIV through male‐male sex (0.56, 0.35−0.90) and did not share their HIV status with HCPs (0.64, 0.42−0.96) were less likely to be refused treatment. Those who did not maintain ART adherence (10.66, 1.16−98.20), sought care at an outpatient clinic before the COVID‐19 pandemic (1.74, 1.00−3.00) and received care at a surgical department (2.10, 1.23−3.60) were more likely to report treatment refusal.

**Table 2:** Correlates of treatment refusal at the most recent outpatient visit among people with HIV (*N* = 502)

	Experienced refusal Did not experience refusal		Multivariable^b^	
	*N* = (212, 42.2%)	*N* = (290, 57.8%)	*aOR (95% CI)*	*p*
**Socio‐demographic characteristics**				
Age, years old				
<30	62 (29.2%)	134 (46.2%)	**0** **.43 (0.25, 0.73)**	***0.002***
30–39	96 (45.3%)	110 (37.9%)	0.81 (0.49, 1.35)	0.42
≥40	54 (25.5%)	46 (15.9%)	Ref	0.002
Gender				
Men	189 (89.2%)	266 (91.7%)	Ref	*0.57*
Women	6 (2.8%)	6 (2.1%)	0.76 (0.36, 1.63)	*0.48*
Transgender	17 (8.0%)	18 (6.2%)	0.45 (0.10, 2.04)	*0.30*
Marital status				
Single	115 (54.2%)	154 (53.1%)	Ref	
In a relationship or married	97 (45.8%)	136 (4.9%)	1.28 (0.86, 1.91)	*0.22*
Education				
College degree or higher	164 (77.4%)	235 (81.0%)	Ref	
Below college degree	48 (22.6%)	55 (19.0%)	0.91 (0.52, 1.60)	*0.74*
Monthly income (CNY)				
<5000	96 (45.3%)	109 (37.6%)	Ref	*0.57*
5000–10,000	73 (34.3%)	113 (39.0%)	1.31 (0.73, 2.37)	*0.36*
>10,000	43 (20.4%)	68 (23.4%)	1.04 (0.61, 1.79)	*0.88*
Occupation				
Student	13 (6.1%)	24 (8.3%)	Ref	*0.97*
Manual labourers	53 (25.0%)	58 (20.0%)	1.20 (0.44, 3.23)	*0.72*
Mental labourers	115 (54.3%)	170 (58.6%)	1.15 (0.59, 2.25)	*0.67*
Unemployed or other	31 (14.6%)	38 (13.1%)	1.08 (0.59, 2.00)	*0.80*
**HIV‐related characteristics**				
Transmission route				
Male‐male sex	154 (72.6%)	247 (85.2%)	**0.56 (0.35, 0.90)**	***0.02***
Male‐female sex or other	58 (27.4%)	43 (14.8%)	Ref	
Years since HIV acquisition				
<3	78 (36.8%)	113 (39.0%)	1.06 (0.66, 1.71)	*0.80*
≥3, <6	79 (37.3%)	106 (36.6%)	0.97 (0.58, 1.61)	*0.89*
≥6	55 (25.9%)	71 (24.4%)	Ref	*0.94*
HIV viral load				
Undetectable	181 (85.4%)	234 (80.7%)	*Ref*	
Detectable or unknown	31 (14.6%)	56 (19.3%)	1.41 (0.81, 2.47)	*0.22*
Maintaining ART adherence				
No	5 (2.4%)	1 (0.3%)	**10.66 (1.16, 98.20)**	***0.04***
Yes	207 (97.6%)	289 (99.7%)	Ref	
**Experience of most recent outpatient visit**				
Shared HIV‐positive status to HCP				
Yes	85 (40.1%)	79 (27.2%)	Ref	
No	127 (59.9%)	211 (72.8%)	**0.64 (0.42, 0.96)**	***0.03***
Time of most recent outpatient visit^a^				
Before 23 January 2020	41 (19.3%)	29 (10.0%)	**1.74 (1.00, 3.00)**	***0.04***
23 January 2020 and later	171 (80.7%)	261 (90.0%)	Ref	
Location of most recent outpatient visit				
Eastern provinces of China	46 (21.7%)	104 (35.9%)	0.71 (0.36, 1.39)	*0.31*
Western provinces of China	27 (12.7%)	35 (12.1%)	Ref	*0.28*
Southern provinces of China	14 (6.6%)	16 (5.5%)	1.00 (0.38, 2.63)	*1.00*
Northern provinces of China	77 (36.3%)	88 (30.3%)	1.19 (0.61, 2.30)	*0.61*
Central provinces of China	48 (22.6%)	47 (16.2%)	1.22 (0.60, 2.47)	*0.59*
Type of hospital for most recent visit				
Hospital specialized in HIV care	23 (10.8%)	37 (12.8%)	0.91 (0.49, 1.68)	*0.75*
Other hospital	189 (89.2%)	253 (87.2%)	Ref	
Department of most recent outpatient visit				
Internal medicine	51 (24.1%)	99 (34.1%)	Ref	***0.003***
Surgery	73 (34.4%)	51 (17.6%)	**2.10 (1.23, 3.60)**	***0.01***
Ear, Nose and Throat	42 (19.8%)	69 (23.8%)	0.83 (0.50, 1.40)	*0.48*
Dermatology or other	46 (21.7%)	71 (24.5%)	1.06 (0.61, 1.86)	*0.83*
Gender of most recent outpatient doctor				
Men	144 (67.9%)	179 (61.7%)	Ref	
Women	68 (32.1%)	111 (38.3%)	1.12 (0.73, 1.71)	*0.56*
Transgender	0	0	0	0

Italic values represent *p*‐values obtained from statistical tests. Bold values indicate statistically significant results (*p* < 0.05).

Abbreviations: aOR, adjusted odds ratio; CI, confidence interval; HCPs, healthcare providers; 1 CNY ≈ 0.14 USD, 22nd October 2024.

^a^ The Chinese government announced the COVID‐19 outbreak in China on 23 January 2020.

^b^ Backward stepwise approach was used in multivariable logistic regression.

As illustrated in Table 3, 29% (262/902) of survey respondents reported having received inpatient medical care. Approximately 63% (165/262) of these respondents indicated they had been refused treatment by HCPs. Among those who were not refused treatment, receiving care from non‐stigmatizing and non‐discriminating HCPs and having an undetectable HIV viral load were identified as equally important factors (30%, 14/47) (Figure 2b). In the multivariable logistic model (Table 3), those who received medical care in central provinces of China (2.60, 1.07−6.31) and who were treated by male doctors (2.31, 1.27−4.22) were more likely to report treatment refusal. The results of the univariable analysis remained consistent and are presented in Tables S4 and S5.

**Table 3:** Correlates of treatment refusal at the most recent inpatient visit among people with HIV (*N* = 262)

	Experienced refusal	Did not experience refusal	Multivariable^b^	
	*N* = (165, 63.0%)	*N* = (97, 37.0%)	*aOR (95% CI)*	*p*
**Socio‐demographic characteristics**				
Age, years old				
<30	43 (26.1%)	37 (38.1%)	0.45 (0.19, 1.04)	*0.06*
30−39	76 (46.1%)	40 (41.2%)	0.92 (0.44, 1.93)	*0.82*
≥40	46 (27.9%)	20 (20.6%)	Ref	*0.10*
Gender				
Men	145 (87.9%)	86 (88.7%)	Ref	*0.81*
Women	7 (4.2%)	3 (3.1%)	1.23 (0.43, 3.54)	*0.71*
Transgender	13 (7.9%)	8 (8.2%)	1.91 (0.28, 13.17)	*0.51*
Marital status				
Single	92 (55.8%)	48 (49.5%)	Ref	
In a relationship or married	73 (44.2%)	49 (50.5%)	1.38 (0.78, 2.44)	*0.27*
Education				
College degree or higher	128 (77.6%)	76 (78.4%)	Ref	
Below college degree	37 (22.4%)	21 (21.6%)	1.21 (0.55, 2.67)	*0.64*
Monthly income (CNY)				
<5000	70 (42.4%)	42 (43.3%)	Ref	*0.82*
5000–10,000	61 (37.0%)	35 (36.1%)	1.01 (0.42, 2.46)	*0.98*
>10,000	34 (20.6%)	20 (20.6%)	1.23 (0.55, 2.77)	*0.62*
Occupation				
Student	11 (6.7%)	7 (7.2%)	Ref	*0.45*
Manual labourers	43 (26.1%)	23 (23.7%)	3.39 (0.76, 15.12)	*0.11*
Mental labourers	86 (52.0%)	50 (51.6%)	1.22 (0.49, 3.05)	*0.68*
Unemployment or other	25 (15.2%)	17 (17.5%)	1.59 (0.08, 31.73)	*0.76*
**HIV‐related characteristics**				
Transmission route				
Male‐male sex	115 (69.7%)	72 (74.2%)	0.80 (0.40, 1.60)	*0.53*
Male‐female sex or other	50 (30.3%)	25 (25.8%)	Ref	
Years since HIV acquisition				
<3	53 (32.1%)	36 (37.1%)	0.68 (0.33, 1.40)	*0.30*
≥3, <6	68 (41.2%)	29 (29.9%)	0.51 (0.25, 1.04)	*0.06*
≥6	44 (26.7%)	32 (33.0%)	Ref	*0.17*
**HIV viral load**				
Detectable or unknown	21 (12.7%)	21 (21.6%)	Ref	
Undetectable	144 (87.3%)	76 (78.4%)	1.89 (0.93, 3.70)	*0.08*
Maintaining ART adherence				
No	1 (0.6%)	1 (1.0%)	1.59 (0.08, 31.73)	*0.76*
Yes	164 (99.4%)	96 (99.0%)	Ref	
**Experience of most recent inpatient visit**				
Time of most recent inpatient visit^a^				
Before 23 January 2020	44 (26.7%)	30 (30.9%)	0.70 (0.37, 1.35)	*0.29*
23 January 2020 and later	121 (73.3%)	67 (69.1%)	Ref	
Location of most recent outpatient visit				
Eastern provinces of China	31 (18.8%)	30 (30.9%)	0.92 (0.40, 2.11)	*0.83*
Western provinces of China	19 (11.5%)	19 (19.6%)	Ref	***0.02***
Southern provinces of China	11 (6.7%)	3 (3.1%)	2.01 (0.91, 4.42)	*0.08*
Northern provinces of China	61 (37.0%)	30 (30.9%)	3.51 (0.82, 14.98)	*0.09*
Central provinces of China	43 (26.1%)	15 (15.5%)	**2.60 (1.07, 6.31)**	***0.03***
Type of hospital for most recent visit				
Hospital specialized in HIV care	29 (17.6%)	23 (23.7%)	0.53 (0.26, 1.11)	*0.09*
Other hospital	136 (82.4%)	74 (76.3%)	Ref	
Department of most recent outpatient visit				
Internal medicine	42 (25.5%)	21 (21.6%)	Ref	*0.16*
Surgery	33 (20.0%)	32 (33.0%)	1.23 (0.46, 3.31)	*0.68*
Ear, Nose and Throat	18 (10.9%)	13 (13.4%)	2.26 (1.01, 5.02)	*0.05*
Dermatology or other	33 (20.0%)	31 (32.0%)	1.90 (0.93, 3.89)	*0.08*
**Gender of most recent outpatient doctor**				
Men	134 (81.2%)	65 (67.0%)	**2.31 (1.27, 4.22)**	***0.01***
Women	31 (18.8%)	32 (33.0%)	Ref	
Transgender	0	0	0	0

Italic values represent *p*‐values obtained from statistical tests. Bold values indicate statistically significant results (*p* < 0.05).

Abbreviations: aOR, adjusted odds ratio; CI, confidence interval; HCPs, healthcare providers; 1 CNY ≈ 0.14 USD, 22nd October 2024.

^a^ The Chinese government announced the COVID‐19 outbreak in China on 23 January 2020.

^b^ Backward stepwise approach was used in multivariable logistic regression.

### 3.2 Contributing factors and potential solutions to treatment refusal

In the interviews, PWH and HCPs in infectious and non‐infectious disease departments were asked to identify the factors that contribute to treatment refusal and suggest potential solutions. PWH identified HIV stigma and discrimination by HCPs, inadequate communication about undetectable HIV viral loads being able to prevent HIV transmission and negligence by health authorities as factors that contributed to treatment refusal (Table S6). Negligence by health authorities was primarily experienced when PWH lodged complaints about treatment refusal with local government offices which were subsequently ignored. HCPs identified fear of acquiring HIV, limited knowledge about HIV and HIV post‐exposure prophylaxis (PEP), limited experience caring for PWH and lack of trust in hospital support if they acquired HIV at work as the primary factors contributing to refusing treatment to PWH in non‐infectious disease departments.

Participants proposed a number of potential solutions to address treatment refusal (Table S4). Maintaining undetectable HIV viral loads and improving communication skills were identified as key strategies by both PWH and HCPs. HCPs who worked in infectious disease departments and PWH stressed the importance of physicians keeping up to date with knowledge about HIV care. Additional education for HCPs about PEP, increased remuneration for medical procedures that put HCPs at risk of HIV exposure, adequate employment protections and compensation for occupational acquisition of HIV, and the establishment of specialized offices to address treatment refusal were also frequently proposed solutions. Participants emphasized the need for structural interventions, including health authorities establishing a dedicated government department that can utilize resources from other relevant sectors to prevent HCPs from refusing to treat PWH as well as investments in delivering non‐HIV care through hospitals that currently specialize in HIV care.

## 4 DISCUSSION

To the best of our knowledge, this is the first nationwide mixed‐methods study to investigate the prevalence and correlates of treatment refusal among PWH in China. The qualitative and quantitative portions of the study found that being refused treatment for DNRH was a common experience in both inpatient and outpatient settings. This finding is consistent with previous studies conducted in India [21], Iran [8] and the United States [13]. We found that 42% and 63% of PWH had experienced treatment refusal during their most recent outpatient and inpatient care, respectively, a figure considerably higher than that observed in the United States (8%) [13]. This discrepancy may be explained by differences in social acceptance of HIV between China and the United States as well as the potential underestimation of treatment refusal among PWH in the United States.

The age of PWH, gender of HCPs, route of HIV acquisition and disclosure of HIV status to HCPs were significant predictors of treatment refusal, highlighting prevalent social stigma and discrimination by HCPs towards PWH in China. Our findings indicate that PWH who were younger than 30 years old, received care from female HCPs, acquired HIV through male‐male sex and did not share their HIV status with HCPs were less likely to report experiencing treatment refusal. Younger PWH and men who have sex with men may have experienced less treatment refusal because of a heightened awareness of the need to communicate about their undetectable HIV viral loads with HCPs [22]. Wider society in China, including doctors, may exhibit greater tolerance towards younger PWH [23]. Our finding that male HCPs are more likely to refuse treatment to PWH aligns with another study documenting a tendency for men to exhibit greater discrimination against LGBTQ+ and PWH communities compared to women [24]. Such discrimination may be driven by dominant patriarchal discourses [25, 26]. The correlations between the mode of HIV acquisition and treatment refusal, as well as between disclosing HIV status to HCPs and treatment refusal, indicate the widespread stigma and discrimination towards LGBTQ+ and PWH communities within the Chinese context, consistent with a large body of previous literature [27, 28].

Limited knowledge on how to prevent occupational acquisition of HIV and the absence of support from health authorities are also pivotal factors in the refusal of treatment. Our quantitative analysis found receiving care at a surgical department was a significant correlate of treatment refusal. This finding is consistent with another study in China which reported that HCPs working in the surgical units exhibited heightened discrimination towards PWH, driven by the elevated risk of occupational acquisition and concerns about acquiring HIV [11]. Interviews indicated that HCPs working in infectious disease departments had a greater understanding of HIV and PEP, which helped mitigate concerns about acquiring HIV when providing care to PWH. Previous studies have identified several factors that contribute to treatment refusal among HCPs. These include misconceptions about HIV [29], fears of incurability [30], concerns about occupational acquisition [31], lack of knowledge about ways to prevent occupational HIV acquisition [32] and insufficient access to PEP [33]. PEP education and availability should be improved for HCPs, with the aim of reducing fear of occupational HIV acquisition, thus reducing treatment refusal. HIV education for healthcare professionals may also reduce treatment refusal [34, 35, 36].

Institutional support from hospitals may also play an important role in making HCPs feel comfortable caring for PWH [11]. In interviews, HCPs expressed concern that hospitals might dismiss them if they acquired HIV through occupational exposure. In addition to legal protections for HCPs, another practical approach to improving government or hospital support is to increase the salaries of those who care for PWH. It is also important to establish clear policies on care and support for HCPs who have acquired HIV through occupational exposure. In addition, the denial of treatment to PWH has been exacerbated by negligence on the part of health authorities. Interviews with PWH revealed that health authorities often ignore cases of refusal of treatment, even when PWH come to them for help. It is crucial that health authorities strengthen their efforts and ensure that legislation, policies and regulations at the provincial and national level provide guarantees against discrimination based on HIV status.

A novel finding of our study is that insufficient communication regarding undetectable HIV viral loads between PWH and HCPs is a significant factor contributing to treatment refusal. Our survey found that individuals who adhere to ART are less likely to report treatment refusal by HCPs. This may be because ART adherence allows participants to maintain undetectable HIV viral loads, and thereby facilitates communication between PWH and HCPs about how undetectable viral loads can prevent HIV transmission [37, 38]. These findings underscore the importance of ART adherence as well as the need to strengthen communication strategies regarding the protections afforded by virologic suppression. To facilitate effective discussions around undetectable HIV viral loads, it is essential to implement a multifaceted approach that addresses the concerns of both PWH and HCPs. This includes the implementation of educational and training programmes that impart effective communication techniques, the establishment of peer support groups for the purpose of shared learning, role‐playing exercises designed to simulate medical consultations and the provision of accessible information materials within PWH communities [39]. It is important to enhance confidence and self‐advocacy [40], and mobile applications and other technology can facilitate these efforts by offering real‐time health information and communication tools. The implementation of public awareness campaigns can assist in the reduction of stigma and the creation of a supportive communication environment. It is also crucial to provide training for HCPs with the aim of enhancing their comprehension of HIV and their ability to communicate with PWH. The implementation of regular feedback mechanisms and community engagement activities, which involve both PWH and HCPs, may further foster understanding and refine communication strategies.

Spatial‐temporal factors significantly influenced treatment refusal in our study. PWH who sought medical assistance in provinces in central China and those who sought outpatient care before the COVID‐19 pandemic were more likely to encounter treatment refusal. The higher prevalence of refusal in Central China compared with Western China may be attributed to the implementation of regulations in some regions of Western China that protect the medical rights of PWH [41]. Further studies focusing on refusal in different regions of China are warranted. With regard to the timing of medical assistance, the COVID‐19 pandemic may have altered HCPs’ attitudes towards PWH. There was a potential observed increase in mutual consideration between patients and HCPs during and after the pandemic. This enhanced empathy and understanding may have contributed to a greater willingness to provide care for PWH.

We propose specific recommendations to reduce treatment refusal based on our findings. First, it is important for PWH to adhere to ART and achieve virological suppression. It is also crucial for PWH to possess effective communication skills regarding undetectable HIV viral loads when seeking medical care. Second, HCPs should enhance their knowledge of HIV, with particular emphasis on PEP. Third, health authorities should implement policies for HCPs who care for PWH, such as increasing salaries for HCPs who perform procedures which put them at increased risk of HIV acquisition and establishing clear job protection and compensation policies for HCPs who acquire HIV through occupational exposure. Fourth, the government should establish dedicated offices to address treatment refusal based on HIV status and support hospitals that specialize in HIV care to expand their services to include non‐HIV care. Efforts should be made to raise awareness of HIV‐related knowledge with the aim of eliminating stigma against PWH through government communications, public education and social media. Structural interventions, including anti‐discrimination legislation that includes explicit protections for PWH, may help prevent denial of treatment to PWH. Our findings may be applicable to other settings, particularly those where HIV‐related stigma and discrimination persist, medical resources are inadequate and relevant legal frameworks are still underdeveloped.

Our study has several strengths. First, we analysed experiences with treatment refusal using data collected from both PWH and HCPs across a range of provinces in mainland China. Recruited HCPs included both doctors who practice in infectious and non‐infectious disease departments. This diversity in study participants helps enhance generalizability and reduce the social desirability bias that can arise from studies which only involve HCPs who specialize in HIV care. To enhance the reliability and validity of our findings [42], we implemented several strategies to bolster the robustness of our results: (1) We applied rigorous inclusion criteria for participants in the quantitative data analysis, ensuring that only those who completed the survey and correctly answered validation questions were included. (2) We conducted a mixed‐methods study, utilizing qualitative and quantitative approaches to enhance the depth and breadth of our analysis. (3) More than one investigator was involved in the qualitative analysis process, ensuring that any discrepancies were thoroughly examined and discussed. Finally, our study proposes potential solutions to address treatment refusal, providing an evidence base to inform policymakers.

Some limitations should also be noted. China has a healthcare system with unique features, and therefore, our findings may not be generalizable to different settings. Survey respondents were recruited through convenience sampling, which may have introduced selection and volunteer bias into the results. Nevertheless, the socio‐demographic characteristics of survey respondents in our study were consistent with those reported in a national systematic review in China [43]. The survey was conducted online, which prevents us from confirming whether respondents had been diagnosed with HIV. However, the questionnaire was distributed exclusively via “Li Hui Shi Kong,” a WeChat social media account specifically tailored for PWH and managed by an HIV clinician. Participants in the semi‐structured interviews were compensated CNY 50, which may have introduced bias. Because we did not collect identifying information, it is unclear whether interviewees also participated in the quantitative survey. Interviews were intended to complement the survey by offering additional information that the survey could not provide, such as solutions to treatment refusal. A total of 35 PWH were interviewed, whereas the questionnaire was completed by 902 PWH. Thus, if the inclusion of interviewees did occur, it likely had a negligible effect on our findings. Furthermore, qualitative interviews with transgender individuals were not conducted. Transgender PWH may experience unique barriers to care that are influenced not only by the general prejudices associated with HIV but also by specific biases related to their gender identity. Such barriers may include negative attitudes from HCPs, inadequate understanding of transgender health needs and a lack of inclusive healthcare policies. It is our hope that future research will focus on the healthcare issues faced by transgender PWH in China. Finally, the findings of our study indicated that PWH were less likely to report treatment refusal after the COVID outbreak. However, due to the small sample size and the considerable variation in data regarding the pandemic across different provinces in China, we are unable to conduct further analysis to explore the association between different stages of the pandemic and treatment refusal.

## 5 CONCLUSIONS

PWH in China frequently experience treatment refusal for DNRH. PWH who are younger than 30 years old, acquired HIV through male‐male sex, attended hospitals in Western China, received outpatient care after the COVID‐19 pandemic, were on ART and who sought medical services in non‐surgical departments were less likely to report being denied treatment. Systematic measures that include all stakeholders, especially laws and policies that protect against discrimination by HIV status, should be taken to reduce the frequency of treatment refusal experienced by PWH in China.

## COMPETING INTERESTS

We declare no competing interests.

## AUTHORS’ CONTRIBUTIONS

HZ conceived the study and designed the protocol in consultation with YH and XZ. YH, XZ, XF, HL, RB, YD and HZ conducted the study. YH performed the data analysis and drafted the manuscript. LH, XZ, XP, DL, HZ, ZG and HZ critically revised the manuscript.

## FUNDING

This study was supported by the Natural Science Foundation of China Excellent Young Scientists Fund [82022064], Natural Science Foundation of China International/Regional Research Collaboration Project [72061137001], the Shenzhen Science and Technology Innovation Commission Basic Research Program [JCYJ20190807155409373] and the Special Support Plan for High‐Level Talents of Guangdong Province [2019TQ05Y230]. All funding parties did not have any role in the design of the study or in the explanation of the data.

## Supporting information




**Table S1**. Interview questions for people with HIV
**Table S2**. Interview questions for healthcare providers
**Table S3**. Perspectives about treatment refusal from different stakeholders
**Table S4**. Refusal at the most recent outpatient visit and its correlates among people with HIV (*N* = 502)
**Table S5**. Refusal at the most recent inpatient visit and its correlates among people with HIV (*N* = 262)
**Table S6**. Potential factors of, and solutions to treatment refusal within healthcare settings

## Data Availability

Research data are not shared to uphold ethical considerations and honour the commitments made to research participants.

## References

[1] Andersson GZ , Reinius M , Eriksson LE , Veronica S , Farhad ME , Keshab D , et al. Stigma reduction interventions in people living with HIV to improve health‐related quality of life. Lancet HIV. 2020;7(2):e129–e140. 31776098 10.1016/S2352-3018(19)30343-1 PMC7343253

[2] Safreed‐Harmon K , Anderson J , Azzopardi‐Muscat N , Georg M , Behrens N , Antonella d'Arminio M , et al. Reorienting health systems to care for people with HIV beyond viral suppression. Lancet HIV. 2019;6(12):e869–e877. 31776099 10.1016/S2352-3018(19)30334-0

[3] Chiao EY , Coghill A , Kizub D , Valeria F , Ntokozo N , Angela M , et al. The effect of non‐AIDS‐defining cancers on people living with HIV. Lancet Oncol. 2021;22(6):e240–e253. 34087151 10.1016/S1470-2045(21)00137-6 PMC8628366

[4] Yuan T , Hu Y , Zhou X , Luoyao Y , Hui W , Linghua L , et al. Incidence and mortality of non‐AIDS‐defining cancers among people living with HIV: a systematic review and meta‐analysis. EClinicalMedicine. 2022;52:101613. 35990580 10.1016/j.eclinm.2022.101613 PMC9386399

[5] Survival of HIV‐positive patients starting antiretroviral therapy between 1996 and 2013: a collaborative analysis of cohort studies. Lancet HIV. 2017;4(8):e349–e356. 28501495 10.1016/S2352-3018(17)30066-8 PMC5555438

[6] Ekstrand ML , Ramakrishna J , Bharat S , Elsa H . Prevalence and drivers of HIV stigma among health providers in urban India: implications for interventions. J Int AIDS Soc. 2013;16(3 Suppl 2):18717. 24242265 10.7448/IAS.16.3.18717 PMC3833193

[7] Dong X , Yang J , Peng L , Minhui P , Jiayi Z , Zhan Z , et al. HIV‐related stigma and discrimination amongst healthcare providers in Guangzhou, China. BMC Public Health. 2018;18(1):738. 29902990 10.1186/s12889-018-5654-8 PMC6003171

[8] Tavakoli F , Karamouzian M , Rafiei‐Rad AA , Abedin I , Mehrdad F , Mehdi N , et al. HIV‐related stigma among healthcare providers in different healthcare settings: a cross‐sectional study in Kerman, Iran. Int J Health Policy Manag. 2020;9(4):163–169. 32331496 10.15171/ijhpm.2019.92 PMC7182146

[9] Feyissa GT , Abebe L , Girma E , Mirkuzie W . Stigma and discrimination against people living with HIV by healthcare providers, Southwest Ethiopia. BMC Public Health. 2012;12:522. 22794201 10.1186/1471-2458-12-522 PMC3506482

[10] Rueda S , Mitra S , Chen S , David G , Jason G , Lori C , et al. Examining the associations between HIV‐related stigma and health outcomes in people living with HIV/AIDS: a series of meta‐analyses. BMJ Open. 2016;6(7):e011453. 10.1136/bmjopen-2016-011453 PMC4947735 27412106

[11] Li L , Wu Z , Wu S , Yu Z , Manhong J , Zhihua Y . HIV‐related stigma in health care settings: a survey of service providers in China. AIDS Patient Care STDs. 2007;21(10):753–762. 17949274 10.1089/apc.2006.0219 PMC2795451

[12] Sarma P , Cassidy R , Corlett S , Barbra K . Ageing with HIV: medicine optimisation challenges and support needs for older people living with HIV: a systematic review. Drugs Aging. 2023;40(3):179–240. 36670321 10.1007/s40266-022-01003-3 PMC9857901

[13] Schuster MA , Collins R , Cunningham WE , Sally CM , Sally Z , Myra W , et al. Perceived discrimination in clinical care in a nationally representative sample of HIV‐infected adults receiving health care. J Gen Intern Med. 2005;20(9):807–813. 16117747 10.1111/j.1525-1497.2005.05049.x PMC1490199

[14] Han S , Li H , Li K , Wang Z . The development and evaluation of a social media‐based HIV knowledge dissemination platform in China. Int J Nurs Sci. 2023;10:288–293. 37545778 10.1016/j.ijnss.2023.06.003 PMC10401336

[15] Sun Y , Li H , Luo G , Meng X , Guo W , Fitzpatrick T , et al. Antiretroviral treatment interruption among people living with HIV during COVID‐19 outbreak in China: a nationwide cross‐sectional study. J Int AIDS Soc. 2020;23:e25637. 33247541 10.1002/jia2.25637 PMC7645858

[16] Crunenberg R , Charles C , Lallemand A , Laetitia B , Geneviève P , Ethgen O . Interpretative phenomenological analysis of the collaboration among healthcare professionals in the nursing home setting. Explor Res Clin Soc Pharm. 2024;13:100424. 38516547 10.1016/j.rcsop.2024.100424 PMC10955404

[17] Saunders B , Sim J , Kingstone T , Shula B , Jackie W , Bernadette B , et al. Saturation in qualitative research: exploring its conceptualization and operationalization. Qual Quant. 2018;52:1893–1907. 29937585 10.1007/s11135-017-0574-8 PMC5993836

[18] Giraudeau B , Higgins JP , Tavernier E , Ludovic T . Sample size calculation for meta‐epidemiological studies. Stat Med. 2016;35(2):239–250. 26286683 10.1002/sim.6627

[19] Yucun C , Cheng L , Mingyu S , Wei D , Qun Z , Hai S , et al. Survey on the current status of medical treatment for patients with HIV/AIDS in Dalian, 2013∼2014. Prevent Med Forum. 2016;22(04):272–274.

[20] Xu W , Zammit K . Applying thematic analysis to education: a hybrid approach to interpreting data in practitioner research. Int J Qual Methods. 2020;19.

[21] Fauk NK , Ward PR , Hawke K , Lillian M . HIV stigma and discrimination: perspectives and personal experiences of healthcare providers in Yogyakarta and Belu, Indonesia. Front Med. 2021;8:625787. 10.3389/fmed.2021.625787 PMC8149745 34055824

[22] Underhill K , Morrow KM , Colleran C , Holcomb R , Calabrese SK , Operario D , et al. A qualitative study of medical mistrust, perceived discrimination, and risk behavior disclosure to clinicians by U.S. male sex workers and other men who have sex with men: implications for biomedical HIV prevention. J Urban Health. 2015;92(4):667–686. 25930083 10.1007/s11524-015-9961-4 PMC4524849

[23] Govindasamy D , Seeley J , Olaru ID , Alison W , Catherine M , Giulia F . Informing the measurement of wellbeing among young people living with HIV in sub‐Saharan Africa for policy evaluations: a mixed‐methods systematic review. Health Qual Life Outcomes. 2020;18(1):120. 32370772 10.1186/s12955-020-01352-w PMC7201613

[24] Ratcliff J , Lassiter D , Markman D , Celeste S . Gender differences in attitudes toward gay men and lesbians: the role of motivation to respond without prejudice. Pers Soc Psychol Bull. 2006;32:1325–1338. 16963604 10.1177/0146167206290213

[25] Franco‐Rocha OY , Wheldon CW , Osier N , Lett E , Kesler SR , Henneghan AM , et al. Cisheteronormativity and its influence on the psychosocial experience of LGBTQ+ people with cancer: a qualitative systematic review. Psychooncology. 2023;32:834–845. 37025048 10.1002/pon.6133

[26] Gupta M , Madabushi JS , Gupta N . Critical overview of patriarchy, its interferences with psychological development, and risks for mental health. Cureus. 2023;15:e40216. 37435274 10.7759/cureus.40216 PMC10332384

[27] Wang Y , Hu Z , Peng K , Xin Y , Yang Y , Drescher J , et al. Discrimination against LGBT populations in China. Lancet Public Health. 2019;4:e440–e441. 31493836 10.1016/S2468-2667(19)30153-7

[28] Burki TK . Discrimination against people with HIV persists in China. Lancet. 2011;377:286–287. 21322842 10.1016/s0140-6736(11)60079-2

[29] Mahajan AP , Sayles JN , Patel VA , Robert HR , Sharif RS , Daniel JO , et al. Stigma in the HIV/AIDS epidemic: a review of the literature and recommendations for the way forward. AIDS. 2008;22(Suppl 2):S67–79. 10.1097/01.aids.0000327438.13291.62 PMC2835402 18641472

[30] Mohammad Bellal H , Kippax S . Stigmatized attitudes toward people living with HIV in Bangladesh: health care workers' perspectives. Asia Pac J Public Health. 2011;23(2):171–182. 19825839 10.1177/1010539509346980

[31] Rutledge SE , Abell N , Padmore J , Theresa JM . AIDS stigma in health services in the Eastern Caribbean. Sociol Health Illn. 2009;31(1):17–34. 18983418 10.1111/j.1467-9566.2008.01133.x

[32] Lin C , Li L , Wu Z , Sheng W , Manhong J . Occupational exposure to HIV among health care providers: a qualitative study in Yunnan, China. J Int Assoc Physicians AIDS Care (Chic). 2008;7(1):35–41. 17641135 10.1177/1545109707302089 PMC2791920

[33] Allan‐Blitz LT , Mayer KH . Missed opportunities: a narrative review on why nonoccupational postexposure prophylaxis for HIV is underutilized. Open Forum Infect Dis. 2024;11:ofae332. 39086468 10.1093/ofid/ofae332 PMC11289484

[34] Uys L , Chirwa M , Kohi T , Minrie G , Joanne N , Lucia M , et al. Evaluation of a health setting‐based stigma intervention in five African countries. AIDS Patient Care STDs. 2009;23(12):1059–1066. 20025515 10.1089/apc.2009.0085 PMC2832642

[35] Markham C , Baumler E , Richesson R , Parcel G , Basen‐Engquist K , Kok G , et al. Impact of HIV‐positive speakers in a multicomponent, school‐based HIV/STD prevention program for inner‐city adolescents. AIDS Educ Prevent. 2000;12(5):442–454. 11063063

[36] Brown L , Macintyre K , Trujillo L . Interventions to reduce HIV/AIDS stigma: what have we learned? AIDS Educ Prevent. 2003;15(1):49–69. 10.1521/aeap.15.1.49.23844 12627743

[37] Etoori D , Ciglenecki I , Ndlangamandla M , Edwards CG , Jobanputra K , Pasipamire M , et al. Successes and challenges in optimizing the viral load cascade to improve antiretroviral therapy adherence and rationalize second‐line switches in Swaziland. J Int AIDS Soc. 2018;21:e25194. 30350392 10.1002/jia2.25194 PMC6198167

[38] Tzaneva V , Iacob T . A better communication with the patients improves the management of HIV disease: a nonsystematic review. Clujul Med. 2013;86:181–184. 26527943 PMC4462508

[39] Downes R , Foote E . HIV and communication skills for practice. HIV Nursing. 2019;19(4):86–93

[40] Bateganya MH , Amanyeiwe U , Roxo U , Dong M . Impact of support groups for people living with HIV on clinical outcomes: a systematic review of the literature. J Acquir Immune Defic Syndr. 2015;68(Suppl 3):S368–S374. 25768876 10.1097/QAI.0000000000000519 PMC4709521

[41] Yulin set up a hotline to protect the rights of people living with HIV and AIDS patients to seek medical treatment. 2022. http://www.ylsd3yy.com/article/1220412092247. Accessed April 3, 2025.

[42] Lincoln and Guba . Lincoln and Guba's Evaluative Criteria (1985). http://www.qualres.org/HomeLinc‐3684.html. Accessed April 3, 2025.

[43] He N , Ding Y , Li J , Shiying Y , Lulu X , Shijie Q , et al. HIV and aging in Mainland China: implications for control and prevention research. Curr HIV/AIDS Rep. 2019;16(6):439–447. 31773404 10.1007/s11904-019-00473-2

