# Supplementary material for: Prevalence, correlates and solutions to people with HIV in China being refused treatment for diseases not related to HIV: a mixed‐methods study
Source: J Int AIDS Soc. 2025 Apr 11;28(4):e26443. doi: 10.1002/jia2.26443 (PMC11986370; doi:10.1002/jia2.26443)
Supplement: Supplementary file 1 — Table S1. Interview questions for people with HIV Table S2. Interview questions for healthcare providers Table S3. Perspectives about treatment refusal from different stakeholders Table S4. Refusal at the most recent outpatient visit and its correlates among people with HIV (N = 502) Table S5. Refusal at the most recent inpatient visit and its correlates among people with HIV (N = 262) Table S6. Potential factors of, and solutions to treatment refusal within healthcare settings [file JIA2-28-e26443-s001.docx]

**Supplementary Table S1. Interview questions for people with HIV**

| **ID** | **Questions** |
| --- | --- |
| 1 | When were you diagnosed with HIV? Could you please share how you contracted the HIV? What are your current HIV viral loads? |
| 2 | Since acquiring HIV, have you experienced any discomfort unrelated to the HIV? Additionally, have you ever been diagnosed with any conditions not related to HIV? Have you undergone any surgical procedures? |
| 3 | When you felt discomfort, did you seek medical attention? If so, what type of hospital do you typically visit for issues unrelated to HIV? Do you prefer hospitals that specialised in HIV care, or do you go to other facilities? |
| 4 | Did you voluntarily share your HIV-positive status to your doctor? If so, could you explain your reasoning? |
| 5 | Do you perceive sharing your HIV-positive status to your doctor as a psychological burden? Are you concerned that your doctor might discriminate against you or withhold care upon learning of your HIV status? Do you fear that this could lead to negative consequences for you? |
| 6 | What has been your previous experience with healthcare providers? Have you ever been denied treatment during an outpatient visit? |
| 7 | Have you ever faced denial of treatment while an inpatient? |
| 8 | Do you believe it is common for people with HIV to experience unfair treatment when seeking medical care? How do you feel about the medical dilemmas faced by people with HIV? |
| 9 | In your opinion, are there specific reasons why individuals with HIV might be more likely to be denied medical care? What recommendations would you suggest for improving access to healthcare for people with HIV? |

PWH=People with HIV.

**Supplementary Table S2. Interview questions for healthcare providers**

| **ID** | **Questions** |
| --- | --- |
| 1 | What is your age? What is your highest level of education, and what was your major? |
| 2 | Have you ever treated patients with HIV? If so, could you share your thoughts and actions when encountering people with HIV (PWH)? |
| 3 | Would you treat HIV-positive patients differently compared to those without HIV? If so, what would be the difference? |
| 4 | If you were to encounter a patient with HIV during your future outpatient or inpatient visits, how would you respond? |
| 5 | Do you believe that people with HIV are more likely to be denied medical care in general hospitals compared to those that specialised in HIV care? What are your reasons for this belief? |
| 6 | How do you feel about the refusal of treatment towards HIV-positive individuals? Have you witnessed this occurring in your surroundings? What do you think contributes to this situation? |
| 7 | Do you believe that individuals with HIV should have the right to receive regular medical care? Why do you think that is? |
| 8 | In your opinion, what is the most significant reason why patients with HIV are more likely to experience treatment refusal? |
| 9 | Do you think there are specific reasons why healthcare professionals might deny medical care to individuals with HIV? What suggestions do you have for reducing treatment refusals for people with HIV? |

HCPs=Health care providers, PWH=People with HIV.

**Supplementary questionnaire:**

**Survey on the Current Medical Care Situation for People with HIV**

**Introduction**

Greetings! We are investigators from Sun Yat-sen University (SYSU) and we are surveying to understand the healthcare experiences of people with HIV (PWH). The insights from the study will help enhance the quality of medical care.

Our study has received ethical approval from the SYSU Ethics Committee. The questionnaire focuses on your physical condition and medical experiences since being diagnosed with HIV. This survey guarantees anonymity, and all data will be kept confidential. Your personal information will not be shared on any platform, nor with any third parties. Future reports stemming from this survey will not include any personal information. You can quit the survey at any time.

Please complete the questionnaire with confidence, and we sincerely appreciate your support!

**HIV and Demographic Information**

1. What is your current HIV status? [Single choice question] *

○ HIV positive, with detectable viral loads

○ HIV positive, with undetectable viral loads

○ HIV positive, but the viral loads are unclear

○ HIV negative (Skip to the end and submit)

○ Unknown (Skip to the end and submit)

2. Which option best describes your gender? [Single choice question] *

○ Man (both physiologically and psychologically)

○ Woman (both physiologically and psychologically)

○ Physiologically woman, psychologically man

○ Physiologically man, psychologically woman

3. The route through which you were acquired with HIV is: [Single choice question] *

○ Heterosexual transmission

○ Homosexual transmission

○ Blood product transfusion

○ Intravenous drug use

○ Mother-to-child transmission

○ Unclear

4. Please fill in the blank: The date of your HIV diagnosis was: *

5. Please fill in the blank with your date of birth: *

6. What is your educational qualification? [Single choice question] *

○ College degree or higher

○ Below college degree

7. Your monthly income in the past year: [Single choice question] *

○ 0-5000 CNY

○ 5000-9999 CNY

○ 10000 CNY and above

8. Your current occupation is: [Single choice question] *

○ Leader with decision-making and management authority

○ Professional technician

○ Institutional administrative staff

○ Service personnel

○ Agricultural, forestry, animal husbandry, fishery production and auxiliary staff

○ Product manufacturing workers/transportation equipment operators

○ Military

○ Student

○ Other, please specify _________________ *

9. What is your marital/partner relationship status? [Single choice question] *

○ Single

○ Married

○ Cohabiting or in a relationship with the opposite-sex partner

○ Cohabiting or in a relationship with a same-sex partner

○ Divorced/Separated

○ Widowed

○ Other, please specify _________________ *

10. Have you ever had the following diseases? [Multiple choice question] *

○ Cardiovascular diseases

○ Tumors

○ Chronic respiratory diseases

○ Other immunodeficiency diseases

○ Other sexually transmitted diseases

○ Other chronic diseases (please specify) _________________ *

○ COVID-19

○ None of these diseases

11. Do you take antiretroviral therapy (ART) regularly? [Single choice question] *

○ Always (≥95%)

○ Sometimes (50~95%)

○ Rarely (≤50%)

○ Never

**Healthcare Experience**

12. After being diagnosed with HIV, your **most recent outpatient** experience when visiting a **non-infectious disease** clinic (including a dental clinic) was/were: [Single choice question] *

○ HCP asked me to leave and go to specialised/other hospitals. (Skip to 14)

○ None of the above, and I had a smooth experience with visiting clinic

13. What do you think was the main reason why your most recent outpatient visit went very smoothly? [Single choice question] *

○ The HCP does not discriminate against PWH

○ I communicated well with the HCP regarding the low transmission risk of undetectable HIV viral loads

○ The HCP did not know my HIV-positive status

○ I utilised personal connections to get a visit

○ Other, please specify _________________ *

14. After being diagnosed with HIV, did the HCP know about your HIV acquisition in your most recent visit to a **non-infectious disease outpatient** clinic (including a dental clinic)? [Single choice question] *

○ Yes

○ No

15. Please fill in the blank, in which city did you last visit a **non-infectious disease outpatient** clinic (including a dental clinic)? *

16. Please fill in the blank, when was the last time you visited a **non-infectious disease outpatient** clinic (including a dental clinic)? *

17. In which of the following hospitals did you visit a **non-infectious disease outpatient** clinic (including a dental clinic)? [Single choice question] *

○ Hospital specialised in HIV care

○ Other hospital

14. Please fill in the blank, what was the specific department that you last visited in a **non-infectious disease outpatient** clinic (including a dental clinic)?

*

15. What was the gender of the HCP in your most recent visit to a **non-infectious disease outpatient** clinic (including a dental clinic)? [Single choice question] *

○ Man

○ Woman

16. After being diagnosed with HIV, your most recent experience when you needed hospitalisation in **non-infectious disease** department was/were: [Single choice question] *

○ HCP asked me to leave and go to specialised/other hospitals. (Skip to 18)

○ I had a smooth experience with hospitalisation.

17. What do you think was the main reason why your most recent hospitalisation went very smoothly? [Single choice question] *

○ The HCP does not discriminate against PWH

○ I communicated well with the HCP regarding the low transmission risk of undetectable HIV viral loads

○ I was in a medical emergency

○ I utilised personal connections to get hospitalised

○ Other, please specify _________________ *

18. Please fill in the blank, in which city did you last hospitalised in a **non-infectious disease** department? *

19. Please fill in the blank, when was the last time you were hospitalised in a **non-infectious disease** department? *

20. In which of the following hospitals did you last hospitalise in a **non-infectious disease** department? [Single choice question] *

○ Hospital specialised in HIV care

○ Other hospital

21. Please fill in the blank, what was the specific department (**non-infectious disease** department) in your most recent hospitalisation? *

22. What was the gender of the HCP in your most recent hospitalisation in a **non-infectious disease** department? [Single choice question] *

○ Man

○ Woman

**Supplementary Table S3. Perspectives about treatment refusal from different stakeholders**

| **Different** **stakeholders** | **Quotations** |
| --- | --- |
| PWH   - Treatment refusal due to sharing HIV-positive status - Suffered rejection of delivering baby - Treatment refusal due to shared prescription system (sharing HIV-positive status) | *“One time when I went to the nephrology clinic, I shared my HIV to the doctor. The doctor immediately said that you should go to the infectious disease departments instead of here, I said "But I’m not here to see the HIV problem, I’m here to see the kidney problem. However, the doctor did not offer any medical care to me.”* (PWH 1, man, 62 years old, diagnosed in 2012)  *“When I went to the hospital to deliver my baby, no hospital offered care to me once they knew I had HIV. I even could not find a safe hospital to have my baby at the first time. I was very desperate at that time.”* (PWH 5, woman, 36 years old, diagnosed 2014)  *“When I used to see the doctor, I told them I had HIV and I was always suffered treatment refusal because of it. Then, I stopped sharing HIV acquisition to HCPs. What I didn't expect was that sometimes I would be denied care even if I didn't tell the medical staff that I had HIV, and then I realised that doctors could know that I had HIV through the shared prescription system. ” (*PWH 30, man, 42 years old, diagnosed 2017) |
| HCPs working in infectious disease departments   - Indirect rejection with some excuse | *“It is true that PWH sometimes suffered treatment refusal, and usually doctors will excuse themselves on various grounds such as poor medical standards, inadequate sterilisation, and failure to meet the conditions of treatment. There are also PWH who are not rejected but consider themselves rejected, it is the low self-esteem of PWH that makes them oversensitive to the verbal eye physical gestures of others and aggravates the feeling of discrimination.” (*Doctor 1, woman, Infectious disease department, Chief doctor) |
| HCPs working in non-infectious disease departments   - fear of occupational acquisition and no compensation if acquired | *“We generally do not refuse to offer treatment to patients with HIV because it is illegal in China. Nevertheless, some HCPs may refuse to offer medical care to patients, but that phenomenon is not common. Most of their reasons for refusal could be the fear of occupational acquisition, which are worried about by all the HCPs. It’s a very common reason. ”* (Doctor 4, man, Rheumatology department, Resident) |

HCPs=Health care providers, PWH=People with HIV.

| Self-discrimination among PWH   - *The need to reduce PWH’s self-discrimination* - *More communication and understanding between PWH and HCPs* | *‘Often the refusal of treatment for PWH is not all the doctor's fault, the patients themselves are also somewhat self-discriminatory, and their own low self-esteem makes them overly sensitive, even normal remarks and physical gestures from doctors make them feel that the doctors are discriminating against and rejecting them.’ (Doctor 22, woman, Infectious disease department, Chief doctor)* |
| --- | --- |

PWH=People with HIV, HCPs=Health care providers, PEP=Post-exposure prophylaxis, ART=Antiretroviral therapy.

**Supplementary Table S4. Refusal at the most r****ecent outpatient visit and its correlates among people with HIV (N=502)**

|  | Experienced refusal Did not experience refusal | | | Multivariable |  |
| --- | --- | --- | --- | --- | --- |
|  | **N =** **(212, 42.2%)** | **N = (290, 57.8%)** | | ***aOR (95%CI)*** | ***P*** |
| Socio-demographic characteristics |  | |  |  |  |
| Age, years old |  |  | |  |  |
| <30 | 62 (29.2%) | 134 (46.2%) | | **0.39 (0.24, 0.65)** | ***<0.001*** |
| 30-39 | 96 (45.3%) | 110 (37.9%) | | 0.74 (0.46, 1.20) | 0.23 |
| ≥40 | 54 (25.5%) | 46 (15.9%) | | Ref | ***<0.001*** |
| Gender |  |  | |  |  |
| Men | 189 (89.2%) | 266 (91.7%) | | Ref | *0.62* |
| Women | 6 (2.8%) | 6 (2.1%) | | 0.75 (0.38, 1.50) | *0.42* |
| Transgender | 17 (8.0%) | 18 (6.2%) | | 1.06 (0.29, 3.93) | *0.93* |
| Marital status |  |  | |  |  |
| Single | 115 (54.2%) | 154 (53.1%) | | Ref |  |
| In a relationship or married | 97 (45.8%) | 136 (4.9%) | | 1.05 (0.73, 1.49) | *0.80* |
| Education |  |  | |  |  |
| College degree or higher | 164 (77.4%) | 235 (81.0%) | | Ref |  |
| Below college degree | 48 (22.6%) | 55 (19.0%) | | 0.80 (0.52, 1.24) | *0.31* |
| Monthly income (CNY) |  |  | |  |  |
| <5000 | 96 (45.3%) | 109 (37.6%) | | Ref | *0.22* |
| 5000-10000 | 73 (34.3%) | 113 (39.0%) | | 1.39 (0.87, 2.23) | *0.17* |
| >10000 | 43 (20.4%) | 68 (23.4%) | | 1.02 (0.63, 1.66) | *0.93* |
| Occupation |  |  | |  |  |
| Student | 13 (6.1%) | 24 (8.3%) | | Ref | *0.43* |
| Manual laborers | 53 (25.0%) | 58 (20.0%) | | 0.66 (0.29, 1.52) | *0.33* |
| Mental laborers | 115 (54.3%) | 170 (58.6%) | | 1.12 (0.61, 2.05) | *0.71* |
| Unemployed or other | 31 (14.6%) | 38 (13.1%) | | 0.83 (0.49, 1.41) | *0.50* |
| HIV-related characteristics |  |  | |  |  |
| Transmission route |  |  | |  |  |
| Male-male sex | 154 (72.6%) | 247 (85.2%) | | **0.46 (0.30, 0.72)** | ***0.01*** |
| Male-female sex or other | 58 (27.4%) | 43 (14.8%) | | Ref |  |
| Years since HIV acquisition |  |  | |  |  |
| <3 | 78 (36.8%) | 113 (39.0%) | | 0.93 (0.62, 1.41) | *0.75* |
| [3, 6) | 79 (37.3%) | 106 (36.6%) | | 1.03 (0.65, 1.62) | *0.92* |
| ≥6 | 55 (25.9%) | 71 (24.4%) | | Ref | *0.91* |
| HIV viral loads |  |  | |  |  |
| Undetectable | 181 (85.4%) | 234 (80.7%) | | *Ref* |  |
| Detectable or unknown | 31 (14.6%) | 56 (19.3%) | | 1.40 (0.87,2.26) | *0.17* |
| Maintaining ART adherence |  |  | |  |  |
| No | 5 (2.4%) | 1 (0.3%) | | 6.98 (0.81, 60.20) | *0.08* |
| Yes | 207 (97.6%) | 289 (99.7%) | | Ref |  |
| Experience of most recent outpatient visit |  |  | |  |  |
| Shared HIV-positive status to HCP |  |  | |  |  |
| Yes | 85 (40.1%) | 79 (27.2%) | | Ref |  |
| No | 127 (59.9%) | 211 (72.8%) | | **0.60 (0.38, 0.82)** | ***0.003*** |
| Time of most recent outpatient visit^#^ |  |  | |  |  |
| Before 23 Jan 2020 | 41 (19.3%) | 29 (10.0%) | | **2.16 (1.29, 3.61)** | ***0.003*** |
| 23 Jan 2020 and later | 171 (80.7%) | 261 (90.0%) | | Ref |  |
| Location of most recent outpatient visit |  |  | |  |  |
| Eastern provinces of China | 46 (21.7%) | 104 (35.9%) | | 0.57 (0.31, 1.06) | *0.07* |
| Western provinces of China | 27 (12.7%) | 35 (12.1%) | | Ref | *0.02* |
| Southern provinces of China | 14 (6.6%) | 16 (5.5%) | | 1.13 (0.47, 2.72) | *0.39* |
| Northern provinces of China | 77 (36.3%) | 88 (30.3%) | | 1.13 (0.63, 2.04) | *0.67* |
| Central provinces of China | 48 (22.6%) | 47 (16.2%) | | 1.32 (0.70, 2.52) | *0.78* |
| Type of hospital for most recent visit |  |  | |  |  |
| Hospital specialised in HIV care | 23 (10.8%) | 37 (12.8%) | | 0.83 (0.48, 1.45) | *0.52* |
| Other hospital | 189 (89.2%) | 253 (87.2%) | | Ref |  |
| Department of most recent outpatient visit |  |  | |  |  |
| Department of internal medicine | 51 (24.1%) | 99 (34.1%) | | Ref | ***<0.001*** |
| Department of surgery | 73 (34.4%) | 51 (17.6%) | | **2.21 (1.32, 3.70)** | ***0.03*** |
| Ear, Nose and Throat | 42 (19.8%) | 69 (23.8%) | | 0.80 (0.48, 1.31) | *0.37* |
| Dermatology or other | 46 (21.7%) | 71 (24.5%) | | 0.94 (0.55, 1.60) | *0.82* |
| Gender of most recent outpatient doctor |  |  | |  |  |
| Men | 144 (67.9%) | 179 (61.7%) | | Ref |  |
| Women | 68 (32.1%) | 111 (38.3%) | | 1.31 (0.90, 1.91) | *0.15* |

aOR=Adjusted odds ratio, CI=Confidence interval, HCPs=Health care providers, 1 CNY ≈ 0.14 USD, 22nd October 2024

^#^ The Chinese government announced the COVID-19 outbreak in China on 23 Jan 2020.

**Supplementary Table S5. Refusal at the most recent inpatient visit and its correlates among people with HIV (N=262)**

|  | Experienced refusal Did not experience refusal | | | Multivariable |  |
| --- | --- | --- | --- | --- | --- |
|  | **N = (212, 42.2%)** | **N = (290, 57.8%)** | | ***aOR (95%CI)*** | ***P*** |
| Socio-demographic characteristics |  | |  |  |  |
| Age, years old |  |  | |  |  |
| <30 | 43 (26.1%) | 37 (38.1%) | | 0.51 (0.26, 1.00) | *0.05* |
| 30-39 | 76 (46.1%) | 40 (41.2%) | | 0.83 (0.43, 1.58) | 0.56 |
| ≥40 | 46 (27.9%) | 20 (20.6%) | | Ref | 0.11 |
| Gender |  |  | |  |  |
| Men | 145 (87.9%) | 86 (88.7%) | | Ref | *0.89* |
| Women | 7 (4.2%) | 3 (3.1%) | | 1.04 (0.41, 2.60) | *0.94* |
| Transgender | 13 (7.9%) | 8 (8.2%) | | 1.44 (0.29, 7.21) | *0.66* |
| Marital status |  |  | |  |  |
| Single | 92 (55.8%) | 48 (49.5%) | | Ref |  |
| In a relationship or married | 73 (44.2%) | 49 (50.5%) | | 0.78 (0.47, 1.29) | *0.33* |
| Education |  |  | |  |  |
| College degree or higher | 128 (77.6%) | 76 (78.4%) | | Ref |  |
| Below college degree | 37 (22.4%) | 21 (21.6%) | | 0.96 (0.52, 1.75) | *0.88* |
| Monthly income (CNY) |  |  | |  |  |
| <5000 | 70 (42.4%) | 42 (43.3%) | | Ref | *0.99* |
| 5000-10000 | 61 (37.0%) | 35 (36.1%) | | 0.98 (0.50, 1.92) | *0.95* |
| >10000 | 34 (20.6%) | 20 (20.6%) | | 1.03 (0.51, 2.05) | *0.94* |
| Occupation |  |  | |  |  |
| Student | 11 (6.7%) | 7 (7.2%) | | Ref | *0.96* |
| Manual laborers | 43 (26.1%) | 23 (23.7%) | | 1.25 (0.39, 4.02) | *0.71* |
| Mental laborers | 86 (52.0%) | 50 (51.6%) | | 1.22 (0.55, 2.69) | *0.63* |
| Unemployed or other | 31 (14.6%) | 38 (13.1%) | | 1.17 (0.58, 2.37) | *0.66* |
| HIV-related characteristics |  |  | |  |  |
| Transmission route |  |  | |  |  |
| Male-male sex | 115 (69.7%) | 72 (74.2%) | | 0.80 (0.45, 1.41) | 0.43 |
| Male-female sex or other | 50 (30.3%) | 25 (25.8%) | | Ref |  |
| Years since HIV acquisition |  |  | |  |  |
| <3 | 53 (32.1%) | 36 (37.1%) | | 0.63 (0.34, 1.15) | *0.13* |
| [3, 6) | 68 (41.2%) | 29 (29.9%) | | 0.59 (0.31, 1.10) | *0.10* |
| ≥6 | 44 (26.7%) | 32 (33.0%) | | Ref | *0.19* |
| HIV viral loads |  |  | |  |  |
| Detectable or unknown | 21 (12.7%) | 21 (21.6%) | | *Ref* |  |
| Undetectable | 144 (87.3%) | 76 (78.4%) | | 1.90 (0.97, 3.69) | *0.06* |
| Maintaining ART adherence |  |  | |  |  |
| No | 1 (0.6%) | 1 (1.0%) | | 1.71 (0.11, 27.63) | *0.71* |
| Yes | 164 (99.4%) | 96 (99.0%) | | Ref |  |
| Experience of most recent outpatient visit |  |  | |  |  |
| Time of most recent outpatient visit^#^ |  |  | |  |  |
| Before 23 Jan 2020 | 44 (26.7%) | 30 (30.9%) | | 1.23 (0.71, 2.14) | *0.46* |
| 23 Jan 2020 and later | 121 (73.3%) | 67 (69.1%) | | Ref |  |
| Location of most recent outpatient visit |  |  | |  |  |
| Eastern provinces of China | 31 (18.8%) | 30 (30.9%) | | 1.03 (0.46, 2.32) | *0.94* |
| Western provinces of China | 19 (11.5%) | 19 (19.6%) | | **Ref** | ***0.02*** |
| Southern provinces of China | 11 (6.7%) | 3 (3.1%) | | 2.03 (0.94, 4.40) | *0.07* |
| Northern provinces of China | 61 (37.0%) | 30 (30.9%) | | 3.67 (0.88, 15.26) | *0.07* |
| Central provinces of China | 43 (26.1%) | 15 (15.5%) | | **2.86 (1.21, 6.82)** | ***0.02*** |
| Type of hospital for most recent visit |  |  | |  |  |
| Hospital specialised in HIV care | 29 (17.6%) | 23 (23.7%) | | 0.69 (0.37, 1.27) | *0.23* |
| Other hospital | 136 (82.4%) | 74 (76.3%) | | Ref |  |
| Department of most recent outpatient visit |  |  | |  |  |
| Department of internal medicine | 42 (25.5%) | 21 (21.6%) | | Ref | *0.12* |
| Department of surgery | 33 (20.0%) | 32 (33.0%) | | 1.30 (0.55, 3.09) | *0.55* |
| Ear, Nose and Throat | 18 (10.9%) | 13 (13.4%) | | 2.11 (1.11, 4.02) | *0.02* |
| Dermatology or other | 33 (20.0%) | 31 (32.0%) | | 1.88 (0.92, 3.85) | *0.09* |
| Gender of most recent outpatient doctor |  |  | |  |  |
| Men | 134 (81.2%) | 65 (67.0%) | | Ref |  |
| Women | 31 (18.8%) | 32 (33.0%) | | **0.47 (0.26, 0.84)** | ***0.01*** |

aOR=Adjusted odds ratio, CI=Confidence interval, HCPs=Health care providers, 1 CNY ≈ 0.14 USD, 22nd October 2024

^#^ The Chinese government announced the COVID-19 outbreak in China on 23 Jan 2020.

**Supplementary Table S6. Potential factors of, and solutions to treatment refusal within healthcare settings**

| Factors of and solutions to refusal | Quotations |  |
| --- | --- | --- |
| Stigmatisation of HIV   - *HIV-related education for all hospital staff* | *‘They (HCPs) actually discriminated against us, and I could feel the contempt and dislike in their eyes. But we are just patients, not bad people, and we deserve to be treated fairly.’ (PWH 1, man, 62 years old, diagnosed in 2012)*  *‘There is inevitably HIV stigma behind this problem, as doctors do not reject patients with other infectious diseases, but reject HIV-acquired patients, so the stigma issue is still ever present and needs to be addressed. Reducing HIV stigma should start in hospitals, because doctors are the people who know the most about the disease.’*  *(Doctor 8, woman, Infectious disease department, Chief doctor)* | |
| Detectable HIV viral loads and insufficient communication skills between PWH and HCPs   - *PWH maintain HIV medication adherence and undetectable HIV viral loads* - *Improve communication skills* | *“I usually take ART regularly and maintain undetectable HIV viral loads, I shared my HIV to the doctors and told them don’t worry because my viral loads is undetectable and the transmission possibility is very low. At first, the doctors was a little bit hesitated because of my HIV and after my communication regarding undetectable HIV viral loads, he provided the treatment to me.” (PWH 8, man, 34 years old, diagnosed in 2017)* |  |
| Negligence of health authorities   - *A special governmental department should be set to deal with the HIV medical dilemma* - *Improving non-HIV-related departments in hospital specialised in HIV care* | *‘When we were denied medical treatment and complained to the hospital or the government, no one helped us, and we hope that the government has the appropriate department to deal with our dilemmas.’ (PWH 20, man, 27 years old, HIV acquisition since 2019)*  *‘We also hope that some hospital specialised in HIV care can improve their non-infectious disease departments, so that we can see (non-infectious disease) doctors in there, and the doctor in the hospital specialised in HIV care usually will not reject us.’ (PWH 26, woman, 36 years old, HIV acquisition since 2015)* |  |
| HCPs’ fear of being acquiring HIV  • *Improve PWH’ ART adherence and maintain undetectable HIV viral loads*  *• HCPs update HIV-related knowledge*  *• HCPs adopt optimal protection*  *• Rapid HIV testing for all emergency patients* | *‘We are all afraid that we will be acquired with HIV when we treat PWH.’ (Doctor 17, man, department of Nephrology, Attending physician)*  *‘We have a lot of HIV patients every day in our infectious disease department and know more about HIV. Nowadays, ART treatment is very effective* *and viral loads is under control, so we are not so easily acquired with HIV, and there are post-exposure prophylaxis drugs. We do not need to worry too much about occupational HIV acquisition.’ (Doctor 3, woman, Infectious disease department, Chief doctor)*  *‘We also know that doctors are afraid of HIV acquisition, but now U=U already indicates that the transmission probability is very low. Many of us have undetectable HIV viral loads, we hope that doctors are not so afraid, and of course we also hope that they adopt the appropriate protective measures.’ (PWH 18, man, 34 years old, HIV acquisition since 2016)* |  |
| HCPs’ limited knowledge about post-exposure prophylaxis   - *Clear education and procedure of post-exposure prophylaxis for HCPs* | *‘I have heard of post-exposure prophylaxis (PEP), but the process in hospitals is very cumbersome, and I do not know how long I have to wait to take PEP after occupational exposure, and I do not know how effective the drug really is.’ (Doctor 12, man, department of Surgery, Resident)*  *‘Now our hospital is basically equipped with PEP, we know a lot about* *PEP* *as I am working in infectious disease department. In our hospital we usually can take PEP within two hours after occupational exposure. I'm not so sure about other hospitals and other departments though.’ (Doctor 8, woman, Infectious disease department, Chief doctor)* |  |
| Limited trust in hospital’s support among HCPs   - *Elevate the salary for risky medical operations* - *Clear liability determination and compensation mechanism for occupational HIV acquisition* | *‘We will not be paid more for treating HIV patients anyway, and we will be at risk of contracting HIV. Once acquired, it is likely that the hospital will terminate our employment, and then we will not even know what to do with it, not to mention treating patients and saving lives. So I think that treating HIV patients may really bring many bad consequences to ourselves.’ (Doctor 14, man, department of Dentistry, Primary care doctor)*  *‘Many doctors are willing to treat PWH, but they are afraid to do so for fear of losing their job if they get HIV. Therefore, I think hospitals should give some financial support to treat PWH patients and give them some protection so that they can be less worried about treating HIV patients.’ (Doctor 22, woman, Infectious disease department, Chief doctor)* |  |

PWH=People with HIV, HCPs=Health care providers, PEP=Post-exposure prophylaxis, ART=Antiretroviral therapy.
